# Evaluation of cell binding to collagen and gelatin: a study of the effect of 2D and 3D architecture and surface chemistry

**DOI:** 10.1007/s10856-016-5763-9

**Published:** 2016-08-31

**Authors:** Natalia Davidenko, Carlos F. Schuster, Daniel V. Bax, Richard W. Farndale, Samir Hamaia, Serena M. Best, Ruth E. Cameron

**Affiliations:** 1 Department of Materials Science and Metallurgy, University of Cambridge, 27 Charles Babbage Road, Cambridge, CB3 0FS UK; 2 Department of Biochemistry, University of Cambridge, Downing Site, Cambridge, CB2 1QW UK

## Abstract

**Abstract:**

Studies of cell attachment to collagen-based materials often ignore details of the binding mechanisms—be they integrin-mediated or non-specific. In this work, we have used collagen and gelatin-based substrates with different dimensional characteristics (monolayers, thin films and porous scaffolds) in order to establish the influence of composition, crosslinking (using carbodiimide) treatment and 2D or 3D architecture on integrin-mediated cell adhesion. By varying receptor expression, using cells with collagen-binding integrins (HT1080 and C2C12 L3 cell lines, expressing α2β1, and Rugli expressing α1β1) and a parent cell line C2C12 with gelatin-binding receptors (αvβ3 and α5β1), the nature of integrin binding sites was studied in order to explain the bioactivity of different protein formulations. We have shown that alteration of the chemical identity, conformation and availability of free binding motifs (GxOGER and RGD), resulting from addition of gelatin to collagen and crosslinking, have a profound effect on the ability of cells to adhere to these formulations. Carbodiimide crosslinking ablates integrin-dependent cell activity on both two-dimensional and three-dimensional architectures while the three-dimensional scaffold structure also leads to a high level of non-specific interactions remaining on three-dimensional samples even after a rigorous washing regime. This phenomenon, promoted by crosslinking, and attributed to cell entrapment, should be considered in any assessment of the biological activity of three-dimensional substrates. Spreading data confirm the importance of integrin-mediated cell engagement for further cell activity on collagen-based compositions. In this work, we provide a simple, but effective, means of deconvoluting the effects of chemistry and dimensional characteristics of a substrate, on the cell activity of protein-derived materials, which should assist in tailoring their biological properties for specific tissue engineering applications.

**Graphical Abstract:**

## 1 Introduction

The extracellular matrix (ECM) of tissues provides mechanical support for cells and supplies correct biological signals for cell activity [1–4]. When used as cell-delivery vehicles in tissue engineering (TE) applications, biopolymer scaffolds should mimic these ECM functions. Biological performance of three-dimensional (3D) matrices are influenced by several parameters such as the nature and availability of cell binding ligands, the chemico-physical (swelling profiles, degradation rates, etc.) and mechanical properties of the scaffold material and the morphology and spatial characteristics of its 3D structure, including mean pore size, interconnectivity, and homogeneity or anisotropy of inner architecture [3, 5–10]. It is important that the contribution of each of these properties to the overall biological activity of scaffolds is characterised to improve the performance of bioconstructs towards different cell lines.

Over recent years, intensive research has been conducted aimed at creating tailor-made 3D scaffolds. These have been based on collagen (Col) and other biomolecules for a wide variety of tissue repair and regeneration applications including tendon [11], cartilage [12], mammary gland [13], and myocardial tissue [14, 15]. In this work, Col and Gel (Gel) were selected as base proteins for biopolymer scaffolds. Col, in particular fibrillar Type I, is the most abundant constituent of the ECM of many hard and soft tissues in the human body [2, 16–19]. This protein provides both the structural support to resident cells and also important cell surface receptor-recognition motifs that are essential for cell–substrate interaction [20–22]. Gel is produced by heating Col, which unfolds the triple-helical conformation present in Col, with the formation of random-coiled domains [23, 24]. As such, Gel possesses a very similar chemical composition to Col, but a less ordered macromolecular structure. The addition of Gel to Col and the variation in crosslinking status can tailor many important material properties of resultant matrices. These include the dissolution resistance in different biological environments, the swelling characteristics and the mechanical strength [15, 25]. In conjunction with this data, the main objective of this research is to evaluate cell interaction with Col and Gel-based biomaterials with a particular focus on the chemical identity and availability of receptor recognition ligands for cell adhesion. In the literature, many studies of cell attachment to protein-derived matrices ignore the detailed mechanism of binding—be it integrin-mediated or non-specific. Integrins are a class of heterodimeric transmembrane cell receptors, composed of one *α* subunit and one *β* subunit, that mediate cell-cell and cell-ECM interactions [26, 27]. In this work, we have used a range of model cell lines which express different integrins. Using cell adhesion analysis of these cell lines we have probed the nature of the integrin binding sites on our materials as a function of biopolymer composition, degree of crosslinking, and two-dimensional (2D) or 3D architecture of the substrate.

**Fig. 1:**
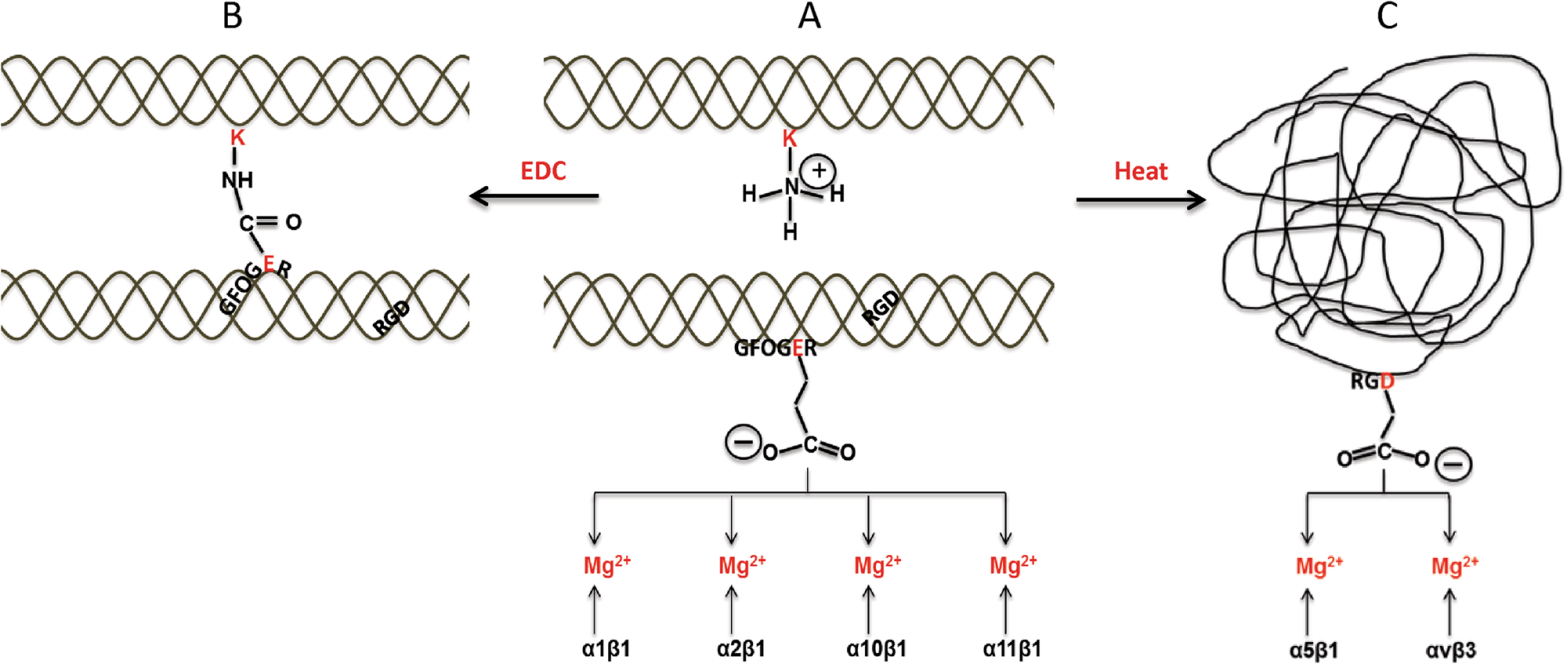
Cellular interactions with Col and Gel; effect of composition and EDC-mediated crosslinking. **a** Two adjacent Col helices are shown; in the first, a lysine amine-containing sidechain is shown, and in the second, the integrin-binding motif GFOGER is located, with its crucial glutamate acidic side chain protruding from the helix. The carboxylate anion is free to co-ordinate a Mg^2+^ ion that is bound to the integrin *α* subunit I domain, so that α1β1, α2β1, α10β1, or α11β1, whichever is expressed on the connective tissue cell surface, can secure cell binding to the matrix. **b** EDC promotes the cross-linking of the glutamate carboxylate group with the adjacent lysine amine group, forming an amide bond between adjacent Col helices. The glutamate sidechain can no longer interact with integrins. **c** Heating the Col unfolds the Col triple helix to yield a disordered, random coil structure, Gel. In the native helical form, the RGD motifs in Col (shown in **a**) are so constrained that they cannot bind integrin. In the unfolded Gel, RGD-containing strands are more flexible, and the aspartate sidechain is free to co-ordinate a Mg^2+^ ion bound in the *β* subunit I-like domain of the integrin. Several integrins can bind RGD motifs in this way, including α5β1 and αVβ3, that are widely expressed in connective tissue cells. Thus, conversion of Col to form Gel by heating switches binding specificity from α1β1, α2β1, α10β1, or α11β1 to α5β1, and αVβ3

In our previous studies, we used UV irradiation and carbodiimide chemistry, based on the reaction with EDC (1-ethyl-3-(3-dimethylaminopropyl-carbodiimide hydrochloride) in the presence of NHS (N-hydroxy-succinimide), to tailor the physical characteristics of scaffolds [15, 25]. EDC crosslinking is a very effective method to increase the mechanical stability and the dissolution resistance of collagenous materials [28–31]. However, this treatment consumes the carboxylate groups on the amino acid side chains of glutamate (E) or aspartate (D). This same chemistry is crucial for ligation by the cell surface integrins [15, 32, 33] as both Col and Gel possess E or D residues in their essential cell-recognition motifs. In Col, these cell binding motifs include the high affinity triple-helical GxOGER sequences (where G is glycine; O is hydroxyproline; R is arginine, and **x** is hydrophobic, exemplified by phenylalanine, F). By contrast Gel contains the linear RGD cell adhesive motif. Col-derived triple-helical ligands such as GxOGER interact with cells via the β_1_-containing integrins, α_1_β_1_, α_2_β_1_, α_10_β_1_ and α_11_β_1_ [20–22, 34]. The main receptor-recognition motif of Gel, RGD, ligates several integrins, but primarily α_5_β_1_ and α_v_β_3_ [24, 35]. The binding of integrins to Col and Gel requires the presence of divalent cations, and Mg^2+^ is the preferred physiological ion [36–38]. Cellular interactions with Col and Gel are schematically presented in Fig. 1. The mechanistic aspects of cell attachment to Col and Gel substrates suggest that changes in composition and in crosslinking status could alter the nature and the availability of cell-recognition sites, thereby affecting the biological reactivity of these materials.

**Fig. 2:**
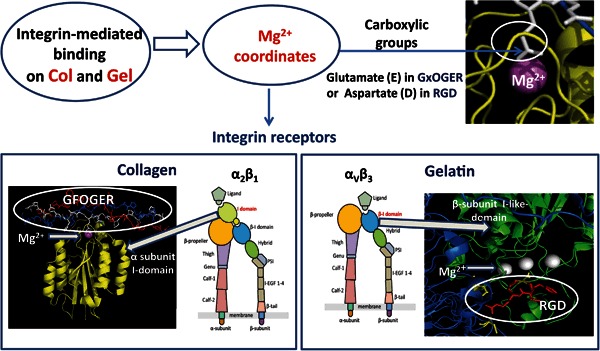
Graphical representation of integrin-mediated adhesion on Col and Gel. Schematics of the integrin structure were adapted from [38]. The crystal structure of the integrin α2 I-domain binding to Col GFOGER was produced from pdb:1DZI and Cyclic RGD binding to the *β*-subunit I-like-domain was produced from pdb:1L5G

Figure 2 represents crystal structures of the integrin domains responsible for the integrin-promoted binding to Col triple helical GFOGER sequences and to Gel cyclic RGD binding motif.

To deconvolute the integrin-based and non-integrin-based cell binding, the adhesion assays were also run in the presence of EDTA (ethylenediaminetetraacetic acid), used to remove divalent cations by chelation. To assess the extent and the nature of cell attachment a series of static adhesion experiments were conducted using different cell lines in the presence of magnesium or EDTA. These were carried out on (a) polystyrene surfaces decorated with Col and Gel from solution (alone or in combination), (b) on 2D thin films of the same compositions, and (c) on 3D scaffolds before and after crosslinking with different EDC concentrations. This experimental approach, based on a systematic increase of the complexity of the system under study, aimed at providing a separate assessment of the influence on cell activity of the chemical identity and the availability/exposure of cell-recognition sequences alone (in coatings), of the influence of the bulk material properties and crosslinking treatments (in films) and of the effect of the complex 3D morphology on the nature and extent of cell-substrate interactions (in scaffolds). Very rigorous washing routines have been applied to films and scaffolds after cell attachment to ensure the removal of non-specifically (weakly) bound cells to substrate in order to minimise the possible cell entrapment within material.

## 2 Materials and methods

### 2.1 Materials

#### 2.1.1 Cell lines

HT1080 (fibroblasts from human sarcoma) cells were obtained from the European Collection of Animal Cell Cultures, Porton Down, UK. C2C12 (mouse myoblast cell line) and C2C12-α2+ (L3 cells), a stably transfected C2C12 with the human integrin α2 subunit, were a gift from Prof D. Gullberg, University of Bergen, Norway. Rugli (derived from a rat glioma) cells were a kind gift from Dr. J. Gavrilovic, University of East Anglia, Norwich, UK.

#### 2.1.2 Materials

Insoluble microfibrillar Col type I (Col) derived from bovine Achilles tendon and Gel (type B from bovine skin, Gel) were purchased from Sigma–Aldrich Co. Ltd. UK. The control triple-helical Col-like peptide GPP10 was synthesized in Farndale lab as described previously [34, 39]. Acetic acid (2 M), EDC and NHS were purchased from Sigma–Aldrich Co. Ltd. UK. Dulbecco Modified Eagles Medium (DMEM), phosphate buffered saline (PBS), Foetal Calf Serum, penicillin, and streptomycin were purchased from Invitrogen Life Sciences (UK). Other commercially available reagents were all analytical grade.

### 2.2 Tested substrates

#### 2.2.1 Monolayer coated surfaces

Col, Gel, and mixed Col/Gel = 50/50 % wt compositions were coated on the surface of Immulon 2HB 96-well plates (Thermo Scientific) by incubating 100 µl/well of 10 µg/ml solution in 10 mM acetic acid containing the appropriate proteins over night at 4 °C. Bovine serum albumin (BSA, Sigma) and triple-helical-like sequences GPP10 were plated in triplicate to act as nonspecific background adhesion controls.

#### 2.2.2 Films

Protein films (Col, Gel, and Col/Gel = 50/50) of ~8 µm thickness were prepared by drying the corresponding 0.5 % (w/v) suspension (Col, Col-Gel) or solution (Gel) of protein in 0.05 M acetic acid directly in Immulon 2HB plates (Thermo Scientific). Suspensions were prepared by swelling Col overnight at 4 °C and then homogenising on ice for 30 min at 13500 rpm using an Ultra-Turrax VD125 (VWR International Ltd., UK). Air bubbles were removed from the suspension by centrifuging at 2500 rpm for 5 min (Hermle Z300, Labortechnik, Germany). Gel solution was prepared at 37–45 °C with stirring for 1 h and then cooled to room temperature. To produce Col-Gel (50/50 %wt.) composition, equal volumes of Col suspension and Gel solution were mixed, homogenised for 15 min and then centrifuged as described above.

#### 2.2.3 Scaffolds

Protein scaffolds (Col, Gel and Col/Gel = 50/50) were obtained by freeze-drying of 1 % (w/v) suspensions (Col, Col-Gel) or 1 % (w/v) Gel solutions in 0.05 M acetic, prepared as described above. These suspensions/solution were poured into silicone rubber trays (Lakeland, UK) and lyophilised in a VirTis adVantage bench-top freeze-drier (Biopharma Process Systems, UK) using a cycle adapted from our previous work [14, 15, 30]. Temperature of −26 °C for freezing and 0 °C for drying under vacuum (less than 100 mTorr) were applied.

### 2.3 Crosslinking

Films and scaffolds were cross-linked (XL) with carbodiimide (EDC) in combination with succinimide (NHS). An EDC concentration of 11.5 mg/ml and molar ratio EDC/NHS/COO^−^(Col) = 5/2/1, was taken as standard (100 %) and was varied from 1 to 200 % of this concentration. After reaction in the corresponding EDC/NHS solution for 2 h at room temperature, the films and the scaffolds were washed thoroughly in deionised water (15 min × 5) and then films were dried in a fume hood while scaffolds were refrozen and re-lyophilized using the previous freeze-drying cycle.

### 2.4 Cell adhesion and spreading

Cell adhesion in the presence of Mg^2+^ (total) and EDTA (non-specific) was assessed calorimetrically through the measurement of lactate dehydrogenase (LDH) activity release from adhered cells into the media.

All cell lines were maintained in a humidified incubator with 5 % CO_2_ at 37 °C in DMEM containing 10 % fetal bovine serum and 1 % streptavidin/penicillin. Prior to cell adhesion experiments, cells were detached from the cell culture flasks with 0.05 % trypsin/0.02 % EDTA (GE Healthcare), washed and re-suspended in serum free DMEM.

#### 2.4.1 Cells adhesion on surfaces and films

Non-specific adsorption to the surfaces/films was blocked with 200 μl per well of bovine serum albumin (BSA, 5 % (w/v) in PBS) for 60 min, and then wells were washed three times with 200 μl of PBS. 100 μl of cell suspension at different concentrations (from 0.5 to 7 × 10^5^ cells/ml in serum free DMEM) containing either 5 mM Mg^2+^ or 5 mM EDTA, were added to the wells and allowed to attach at room temperature for 60 min. The wells were washed with PBS (200 μl × 3) to remove loosely bound cells and then 50 μl of lysis buffer containing 2 % v/v Triton X-100 in distilled water was added for 90 min at room temperature. Subsequently 50 μl of LDH detection substrate (cytotoxicity detection kit (LDH), Roche, Cat. No 11 644 793001) prepared according manufacture instruction, was added and left until color had developed (from 10 to 30 min). The absorbance was read at 490 nm (A_490_) using a Fluostar Optima plate reader (BMG Labtech). Background adhesion was determined on BSA and GPP10 coated plates. Cell adhesion assays were performed in triplicate and values are reported as means ± standard deviations.

Adhesion on films was carried out in the presence and absence of cyclo Arg-Gly-Asp-D-Phe-Val, (cRGD) (Calbiochem, Nottingham, UK, Cat No182015) following the same protocol as above except that cell suspensions containing 5 mM Mg^2+^ and 10 mM cRGD were pre incubated for 15–20 min prior to seeding.

For quantitative analysis of adhesion linear regression calibration curves were constructed from the OD (optical density) vs. initial cell concentration for each experiment. The calibration was obtained by taking 500 µl aliquot of cell suspension at a known cell density and then subsequently serially diluting this from 32 to 64 times depending on the cell density. These known cell number suspensions were centrifuged and the cell pellet lysed by adding 500 μl of buffer containing 2 % v/v Triton X-100 in distilled water for 90 min at room temperature. The cell lysate was vortexed and then and 50 µl aliquots of each solution were pipetted in triplicate on to the same plate corresponding to the cell attachment analysis. After that 50 μl of LDH detection substrate were added to the calibration series at the same time as to the substrates under study and left until color had developed (from 10 to 30 min). The absorbance of this series was read under the same conditions/time as on coated wells.

#### 2.4.2 Cell adhesion on scaffolds

Scaffold discs were cut from the central part of scaffold sheets using a sterile 8 mm biopsy punch (8 mm (d) x 2–3 mm (h), 1.9–2.3 mg) and incubated (6 replicas for each composition/XL condition) with 500 µl of PBS for 1 h in 24-well tissue culture plates (Thermo Scientific). The scaffolds were removed, gently pressed between sheets of filter paper and placed into wells with 500 µl of cell suspension (concentrations from 1 to 5 × 10^5^ cells/ml) in serum free DMEM, containing either 5 mM Mg^2+^ or 5 mM EDTA. These were incubated for 60 min at room temperature to allow cell attachment. The scaffolds were removed, placed in 7 ml tubes and washed with 5 ml of serum free DMEM, containing either 5 mM Mg^2+^ or 5 mM EDTA according to the attachment conditions. Tubes were put on a roller for 15 min and this procedure was repeated 5 times to ensure the complete removal of the media with non-attached or loosely bound cells from the scaffold porous structure. 500 μl of lysis buffer containing 2 % v/v Triton X-100 in distilled water was added for 90 min at room temperature. 50 μl aliquots of lysis solution was pipetted in triplicate into 96 well plate and 50 μl of LDH detection substrate, prepared according manufacture instruction, was added and incubated until color had developed (from 10 to 30 min). The absorbance was read at 490 nm (A_490_) using a Fluostar Optima plate reader (BMG Labtech). For quantitate evaluation of adhesion each experiment was carried out in presence of a calibration series (as described above). Cell adhesion on scaffolds was performed in triplicate and values are reported as means ± standard deviations.

#### 2.4.3 Cell spreading tests

For spreading analysis, 100 μl of cell suspension at 1 × 10^5^ cells/ml containing either 5 mM Mg^2+^ or 5 mM EDTA in serum free DMEM were added to BSA blocked surfaces for 90 min at 37 °C/5 % CO_2_. The cells were fixed by the addition of 9 μl of 37 % (w/v) formaldehyde (final concentration 3.7 %) directly to the cell media for 20 min at room temperature. The samples were washed 3 × 200 μl PBS then viewed using a LEICA DMI6000CS phase contrast microscope fitted with a LEICA DFC340FX camera. Assays were performed in triplicate.

Cell spreading (percentage of spread cells versus total number of cells) was determined by analyzing 12 images per condition and applying the following equation (1):

1 $$ \eqalign{ & {\rm{\% }}\,{\rm{Spread}}\,{\rm{Cells}}\,{\rm{(per}}\,{\rm{image)}} = \\ & \qquad\frac{{{\rm{\# Spread}}\,{\rm{Cells}}}}{{{\rm{\# }}\,{\rm{Total}}\,{\rm{Cells}}\,{\rm{(Spread + Non - Spread}}\,{\rm{Cells)}}}}}$$

The error was determined as the standard deviation between spreading % values calculated from at least three separate experiments, each with triplicate measurements for each experimental condition.

### 2.5 Statistical analysis

Data are expressed as the mean ± standard deviation (SD). Statistical analysis was performed using the two population Student’s *t*-test assuming unequal variances. The significant level (*) was set as *P* ≤ 0.05. (**) indicates *P* ≤ 0.01; (***) indicates *P* ≤ 0.001 and (****) indicates *P* ≤ 0.0001.

## 3 Results

### 3.1 Cell adhesion and spreading on monolayer coated surfaces

Studies were first performed on monolayer coatings of the molecules of interest applied to a polystyrene tissue culture plastic surface. Since only single molecule layers were used, no crosslinking was applied to the molecular surfaces. Testing cell interactions when the material is presented in this form means that the surface is two-dimensional and that bulk mechanical effects such as different stiffnesses are eliminated.

Cell lines selected in this work allow a comparison to be made between the interaction of Col and Gel-based compositions with cells that express Col-binding integrins (HT1080 and L3 expressing α2β1, and Rugli expressing α1β1) and a parent cell line C2C12, which only possess Gel-binding integrins, αvβ3 and α5β1. BSA, frequently used to block any active sites on well surfaces, preventing cells from adhesion to any uncoated plastic, and GPP10 peptide, which adopts a Col-like triple helix [40, 41], but lacks any cell recognition motifs were used as negative control.

#### 3.1.1 Adhesion of different cell lines to monolayer coated surfaces

**Fig. 3:**
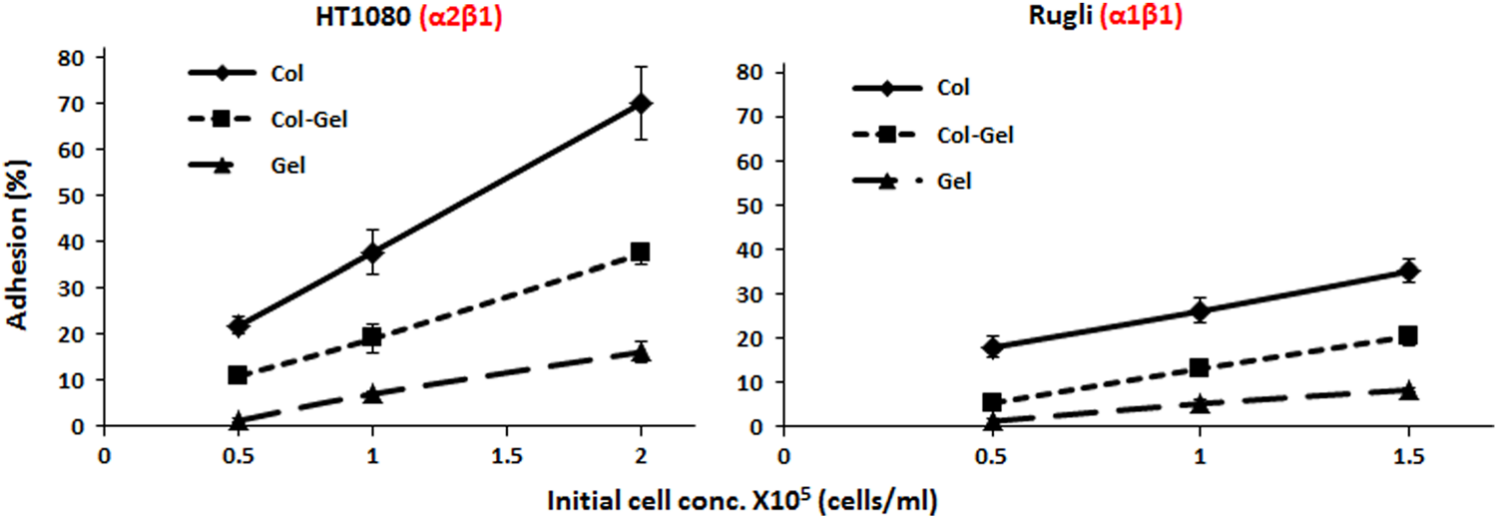
Magnesium dependent adhesion (percentage of adhesion) of HT1080 (left panel) and Rugli (right panel) cells on surfaces of different compositions as a function of initial cell concentration

All cell adhesion tests on coatings were carried out in the presence of calibration solutions (as described in 2.4.1) in the interval of the initial cell concentrations varying from 0.5 to 8 × 10^5^ cells/ml in order to establish the dependence of adhesion percentages on the seeded density. Results revealed that adhesion values, calculated using calibration curves, increased linearly with the seeded cell concentration, in a range from 0.5 to 1.5–2 × 10^5^ cells/ml, for all compositions studied (Fig. 3). At higher initial cell concentrations this linearity was gradually altered, reaching saturation at values higher that 4–5 × 10^5^ cells/ml (data not shown).

**Fig. 4:**
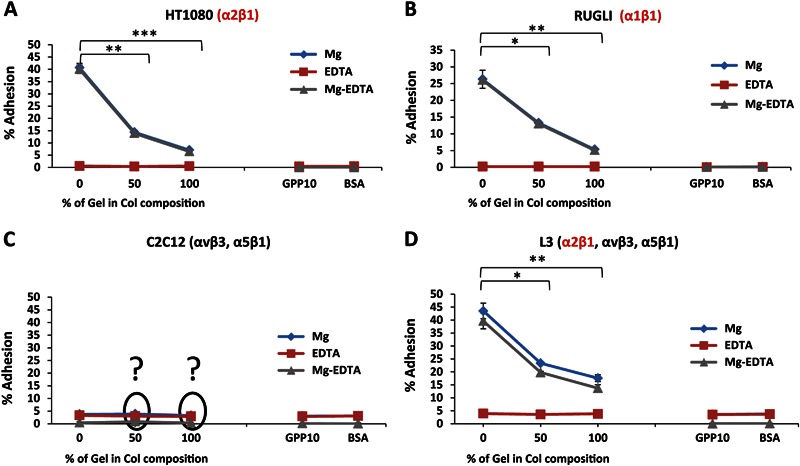
Magnesium-dependent, non-specific (EDTA) and integrin mediated (Mg-EDTA) adhesion profiles of different cell lines as detailed in panels A to D, below, on treated surfaces. Initial cell concentration 1 × 10^5^ cells/ml. * indicates *P* ≤ 0.05, **indicates *P* ≤ 0.01 and *** indicates *P* ≤ 0.001 (*t*-test) against different percentage of Gel in Col composition values

Adhesion profiles of Mg^2+^-dependent (all adhesion), non-specific (EDTA) and only integrin dependent cell attachments on Col and Gel-based substrates are displayed in Fig. 4. These profiles show the cell adhesion percentage values in the linear concentration dependence interval (1 × 10^5^ cells/ml) for all cell lines. It can be observed that for cell lines expressing Col-binding integrins (HT1080, Rugli and L3; Fig. 4a, b, d) all adhesion is integrin-dependent. For these three cell types, the addition of Gel to Col influences adhesion pattern in the same way: adhesion values decrease with the increase of Gel content. This is probably due to a decrease in the density of available integrin-binding sites (triple-helical GFOGER sequences) with the rise of Gel.

In a case of the C2C12 parent cells (expressing only Gel-recognition receptors) no adhesion was observed on Col coatings. Surprisingly, these cells have also not attached to Gel-containing samples (50 % and 100 % Gel, Fig. 4c) in spite of the fact that both compositions possess RGD recognition sequences likely to be revealed in the unfolded Col that are directed to αvβ3 and α5β1 receptors expressed in C2C12. This result suggests that cells do not identify RGD adhesion cues in Gel-based coatings. It seems likely that in creating a monolayer coating of Gel, the conformation of the flat RGD motif is altered, changing its exposure to cell recognition receptors and making it inactive.

Adhesion percentages summarised in Table 1 indicate that on pure Col coatings the adhesion is higher for cells expressing α2β1 integrin (HT1080 and L3) than for Rugli, which express α1β1. These results point to differences in affinity of Col cell-recognition sequences towards these two Col-binding receptors. On mixed compositions (50 % of Gel) and on pure Gel samples, the values were higher for L3 cells (expressing Col and Gel-binding receptors) than for cells possessing only Col-binding integrins (HT1080 and Rugli).

**Table 1:** Adhesion percentage on surfaces of cell lines expressing Col-binding integrins

Adhesion (%)			
Cell concentration 1 × 10^5^ cells/ml			
	Cell line		
	HT1080	Rugli	L3
Col	37.8 ± 4.6	26.2 ± 2.8	42.6 ± 3.0
Col-Gel	11.0 ± 1.1	19.1 ± 3.1	21.1 ± 2.3
Gel	7.1 ± 0.7	5.3 ± 0.7	15.2 ± 1.3

Note: Results are expressed as mean values of three parallel measurements ± standard errors

#### 3.1.2 Spreading of all cell lines on monolayer coated surfaces

**Fig. 5:**
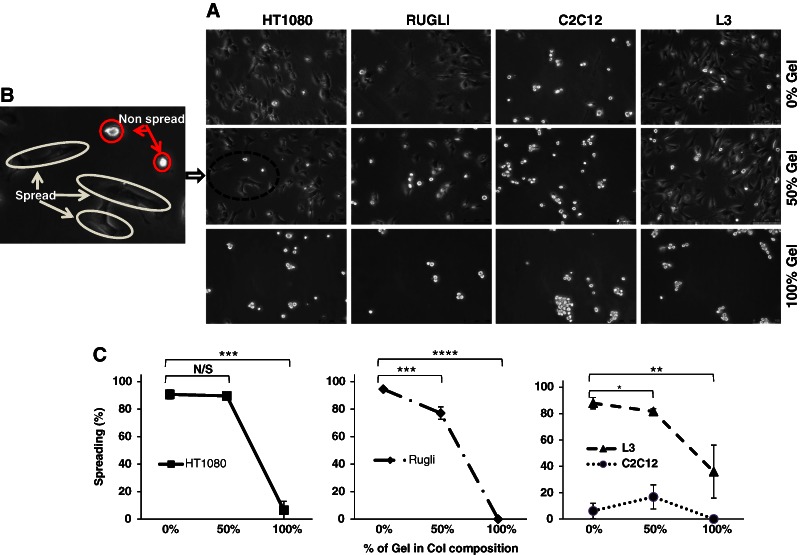
Images of cells that are exposed to Col, Col-Gel, and Gel surfaces in the presence of Mg^2+^ **a**. The enlarged area **b** shows how cells are categorising as spread (large, phase contrast dark) or non-spread (small, phase contrast bright). Quantification is the percentage of cells that are spread **c**, illustrating the effect of composition on the level of spreading of attached cells

Images of the cell spreading of all cell lines in presence of Mg^2+^ are displayed in Fig. 5a. In EDTA containing media, no spreading was detected for any cell line on any surface (data not shown), which is in concordance with the results of adhesion experiments where no attachment was observed for cells incubated in the presence of EDTA.

Results in Fig. 5a show that HT1080, Rugli and L3 cells, expressing Col-binding integrins, are all spread in a similar way on Col-based samples. C2C12 cells, possessing only Gel recognition receptors, were all round-shaped (not spread) on any coatings including pure Gel composition.

Quantification of spreading capacity for cells expressing Col-binding receptors (Fig. 5c) showed a very high level of spreading (between 95 and 90 %) on Col coatings, being lower on mixed Col-Gel samples for L3 (82 %) and especially for Rugli cells (77 %). Statistical analysis confirmed significant differences between spreading values of HT1080, L3 and Rugli cells on Col and Gel coatings and also between pure Col and samples containing 50 % of Gel in case of L3 and Rugli cells.

### 3.2 Cell adhesion on thin films

**Fig. 6:**
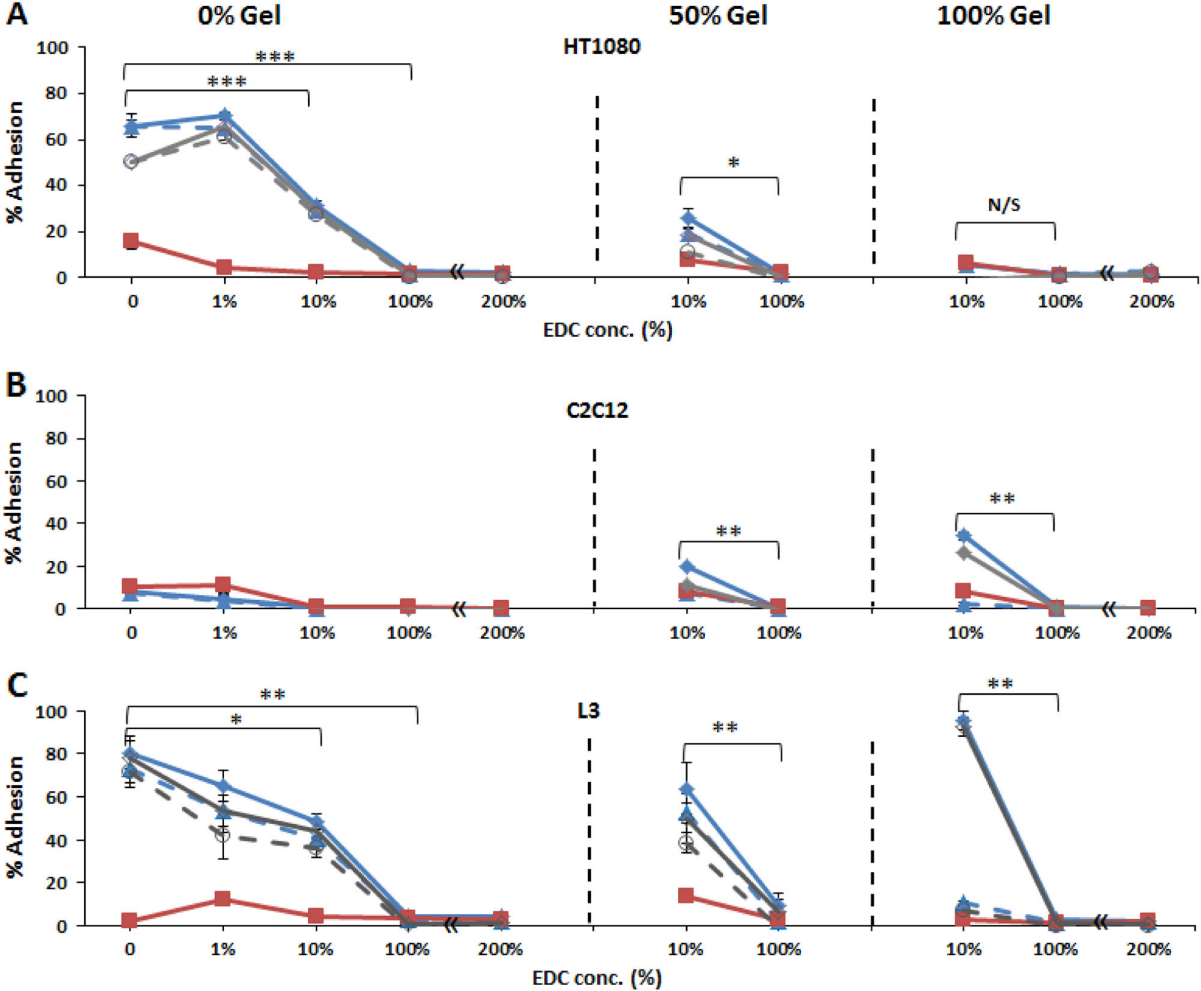
Adhesion (%) of HT1080 **a**, C2C12 **b** and L3 **c** cells on films with different composition and crosslinked status. Initial cell concentration 1 × 10^5^ cells/ml. Full circle (●) with solid line shows Mg^2+^– dependent cell adhesion; triangle (∆) with dashed line shows Mg^2+^– dependent cell adhesion in presence of cRGD; full square (▪) with solid line shows EDTA-dependent adhesion; empty circle (○) with solid line shows only integrin-dependent adhesion (Mg^2+^– EDTA) and empty circle (○) with dashed line shows only integrin-dependent adhesion (Mg^2+^– EDTA) in presence of cRGD. Composition of films and cell line is indicated above each panel.

Having observed the behaviour of the cells on monolayer surfaces, we next applied cells to thin films of the molecules of interest. In this form, the materials presented to the cells are still 2D, but are thick enough to exhibit stiffnesses representative of the bulk materials and molecular conformations unaffected by the underlying substrate. Furthermore, in thin films, the chemical identity and the availability of cell-recognition sequences may be changed not only by composition, but also by alteration in a crosslinking status. Studies on films were carried out in presence and absence of cRGD, a selective antagonist of α_ν_β_3_ and α_v_β_5_ integrins, to establish (a) whether the RGD motif is in a right configuration for cell recognition and (b) if the attachment of C2C12 parent and α2-positive, L3, cells were via RGD binding sequences. For comparison, adhesion of HT1080 cells was also tested on films in presence and absence of cRGD peptide. Fig. 6 shows the adhesion percentages of all cell lines on films with different composition and crosslinking conditions. No results are presented on Non-XL Col-Gel and Gel samples as these compositions were too unstable to resist incubation without partial dissolution and/or detachment from the well surfaces, which may alter the values of cell adhesion.

The results displayed in Fig. 6a show that for HT1080 cells, Mg^2+^-dependent cell adhesion on Col-based scaffold (with and without 50 % of Gel) was due to binding of α2β1 to GxOGER sequences of Col. This process, as expected, was not affected by the presence of the cRGD. No adhesion of HT1080 was detected on Gel films due to the absence of RGD-recognition receptors in this cell line. Conversely, the parent C2C12 cells do not adhere to pure Col films but do show the integrin-mediated attachment to both pure Gel and to the mixed Col-Gel films (Fig. 6b) suggesting that the exposure of RGD motif to cells in Gel-containing films is recognisable by cell surface integrins (unlike Gel-coated surfaces). Moreover, C2C12 attachment to Gel-containing films was completely blocked by the presence of the RGD antagonist, cRGD (Fig. 6b), which confirms that RGD ligand is responsible for cell attachment via αvβ3 and α5β1 integrins. For the L3 cells, which possess α2β1, αVβ3, and α5β1 integrins, the detected Mg^2+^-dependent adhesion on pure Col and Col-Gel was similar to HT1080 and can be attributed almost entirely to binding of α2β1 to GxOGER sequences as binding was largely insensitive to the presence of the cRGD (Fig. 6c). On pure Gel films, the Mg^2+^-promoted adhesion was observed for both L3 and C2C12 cells because of interaction of αvβ3 and α5β1 with RGD ligands. The attachment of both cell lines was abolished by the presence of cRGD, with no difference in the response to EDTA-inhibited samples.

Analysis of the influence of crosslinking on integrin-promoted cell attachment to films showed that adhesion decreases with increase of EDC concentration for all cell lines. This suggests that EDC-mediated treatment may abolish cell adhesion by consuming cell binding sites on Col and Gel-based films.

### 3.3 Cell adhesion on 3D scaffolds

Finally, after considering monolayer coated surfaces and thin films, we applied cells to 3D scaffolds made from the molecules of interest. In this form, the scaffold struts are expected to have similar mechanical properties and molecular conformations as the thin films, but with the added complexity of a 3D porous structure. Cell attachment experiments on scaffolds addressed the influence of both composition and crosslinking (from non-XL to 100 % EDC-treated Col-based samples) on the cell interaction with highly porous 3D substrates.

**Fig. 7:**
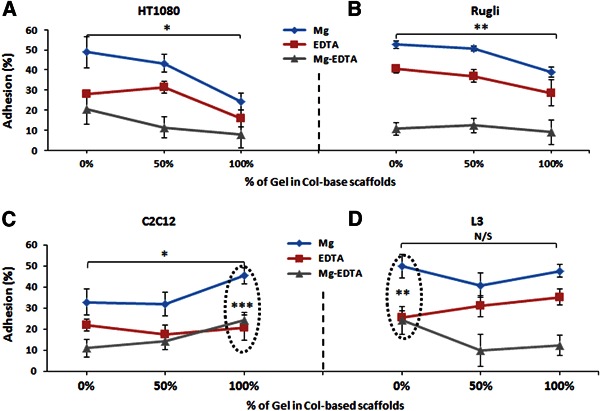
Adhesion (%) of HT1080 **a**, Rugli **b**, C2C12 **c** and L3 **d** cells on 100 % EDC-XL scaffolds of different compositions. Initial cell concentration 5 × 10^5^ cells/ml. N/S indicates no significant differences between values (*P* ≥ 0.05)

The effect of composition may be observed in Fig. 7, where adhesion profiles of 100 % EDC treated pure Col scaffolds with and without addition of different percentages of Gel are displayed. A common feature of all adhesion patterns on scaffolds is a significant level of non-integrin-mediated interaction (in presence of EDTA) between cells and 3D substrates. This is markedly different from the results on monolayer coated surfaces and thin films where non-specific adhesion is consistently low. Mg-dependent adhesion depends on both composition and cell line. Cell adhesion decreases with Gel content for both HT1080 and Rugli cells (Fig. 7a, b), is comparable on Col and Gel scaffolds for L3 (Fig. 7d) and is greatest on Gel scaffolds for C2C12 (Fig. 7c). In the case of L3 cells, Mg^2+^-dependent adhesion is significantly higher than non-specific (EDTA) for pure Col scaffolds (*P* ≤ 0.01). In contrast, Mg^2+^-mediated adhesion of C2C12 parent cells was significantly higher than EDTA-promoted (*P* ≤ 0.001) for Gel samples as a results of the presence of Gel-binding receptors in C2C12.

**Fig. 8:**
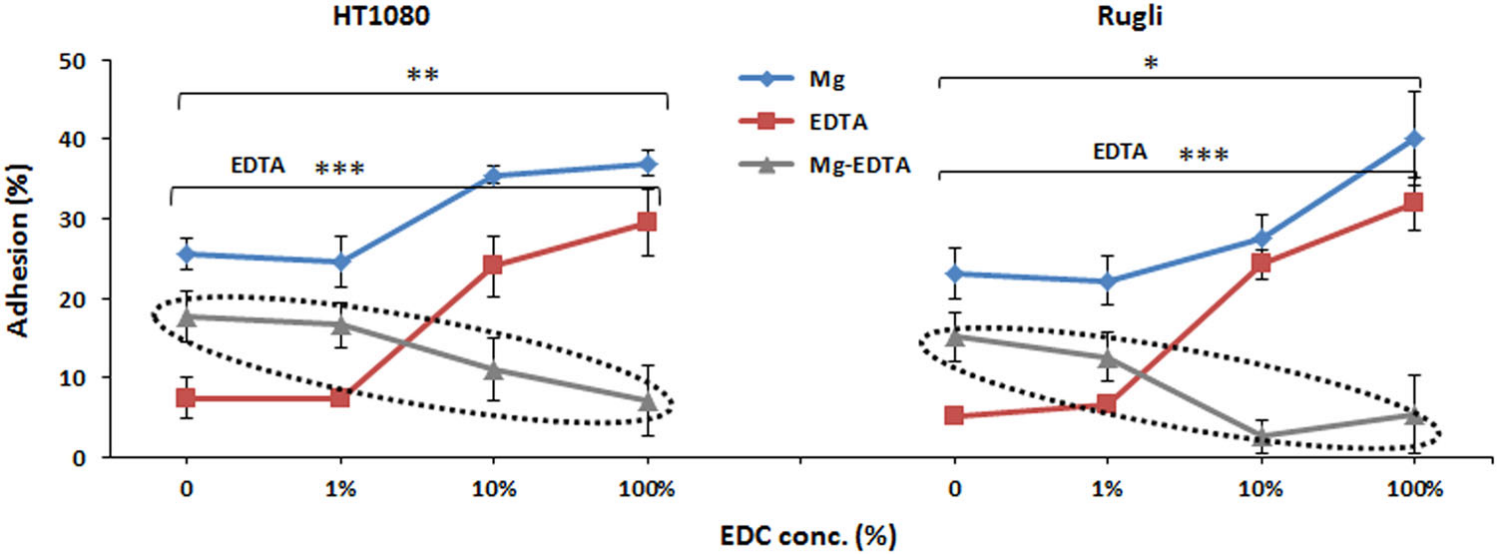
Effect of crosslinking on the adhesion of cells expressing Col-binding integrins (HT1080 and Rugli) on Col scaffolds. Mg indicates total adhesion, EDTA indicates non-specific cell-scaffold interactions and Mg-EDTA shows only integrin-mediated adhesion. Initial cell concentration 5 × 10^5^ cells/ml

Comparison of the effect of crosslinking on adhesion values of HT1080 and Rugli cells on Col scaffolds (Fig. 8) shows that both the total adhesion (Mg^2+^ dependent) and the non-specific, non-integrin promoted (in the presence of EDTA) adhesion significantly increase with crosslinking. However, the integrin-mediated interactions (lines inside dashed circles on Fig. 8) decrease with the increase of crosslinking (in agreement with the results obtained on films) suggesting that EDC crosslinking diminishes the availability of cell-binding ligands on both 2D and 3D substrates.

## 4 Discussion

Cell adhesion is usually the first step in the biological assessment of biomaterials aimed at TE applications. Adhesion studies were carried out on Col and Gel-based substrates with different 2D and 3D architecture in order to establish the influence of composition and crosslinking treatment on the extent and nature of attachment of cell lines expressing different matrix-binding receptors. Samples were studied in the form of monolayer coated surfaces, thin films, and scaffolds to assess the effects of bulk properties and of 2D and 3D presentation.

### 4.1 Adhesion and spreading on monolayer coated surfaces

Adhesion and spreading on monolayer coated surfaces prepared with the same protein content as films and scaffolds provide the possibility of creating the same assembly of integrin recognition sequences as in the scaffold struts. This in turn allows the influence of the chemical identity of ligands and availability/accessibility of these cell binding motifs on cell-substrate interactions to be assessed without the interferences from physical properties and/or the complex 3D architecture.

Adhesion profiles for cells on surfaces revealed that the addition of Gel to Col composition caused a decrease in the ability of cell lines expressing Col-binding integrins to attach to the substrate. This may be explained by a decrease in the availability of GxOGER and an increase in the availability of RGD when the base protein layer is changed from Col to Gel. The decrease in GxOGER ligand density consequently diminishes the number of cell-recognition cues required for cell attachment via Col-binding receptors (α2β1 and α1β1). Adhesion on pure Col coatings was higher for HT1080 and L3 cells, both expressing α2β1 integrin, than for the Rugli cell line, which expresses α1β1 receptors. This may be attributed to differences in the affinity of Col GxOGER ligands towards α2β1 and α1β1 integrins, reported in [42]. The lack of adhesion of C2C12 parent cells to Gel surfaces may be the result of configurational changes in RGD sequences, most probably due to their interaction with surfaces. It seems that a flattened topology of this linear motif on the plastic substrate induces some kind of bond formation between RGD and the surface, which may alter the correct exposure of this motif to cell receptors, suggested to require RGD presentation in a flexible loop [43]. This may explain the lack of attachment of C2C12 on Gel surfaces. This is an important potential limitation of the use of monolayer surface coatings in cell binding assays.

Spreading assays were performed to assess the ability of bound cells to spread as a result of the correct stimulation of certain signaling pathways after attachment. This provides the alternative way of evaluating the “quality” of adhesion: be it integrin-mediated (leading to spreading) or non-specific (no spreading, no further cell activity). These assays were performed in serum-free media to prevent cell adhesion to serum containing proteins such as vitronectin and fibronectin, which may alter spreading patterns. The evaluation of spreading was based on the analysis of cell shape (extended vs. round-shaped) according to a traditional view of cell spreading. The overall cellular surface coverage was not taken into account as it reflects more the degree of cell attachment than cell spreading ability. Results confirmed the importance of integrin-specific interactions on cell activity: spreading of cells expressing Col-binding receptors (HT1080, Rugli and L3) was very high on Col-based surfaces, which points to the correct stimulation of cell attachment mechanisms in these systems. Lack of spreading of C2C12 cells on Gel surfaces (only round cells) is in agreement with the absence of integrin mediated adhesion on Gel samples.

### 4.2 Adhesion on thin films

Adhesion tests on films were performed to assess the cell-scale properties of 3D matrices without interference from the complex 3D morphology of a scaffold. In thin films, both composition and crosslinking were systematically modified to evaluate the impact of these changes on the biological activity of the resultant systems. All films were of ~8 µm thickness, which guaranteed the separation (for several layers) of cell-recognition ligands from the plate surface in order to ensure that the conformation of cell-binding sequences exposed to cells might not be compromised by their interactions with the surface. As such we anticipated the appropriate exposure of both Col and, especially, Gel-binding ligands to cells. The response of C2C12 myoblasts, α2 positive C2C12 (L3), and HT1080 cells on films containing Col, Gel, and a combination of both showed strong influence on cell adhesion of the alteration in the availability of binding sites, induced by changes in composition and the extent of crosslinking. For Col-based compositions, the trends in the adhesion results on films are in agreement with the trends found on the corresponding surfaces for all the cell lines studied. On mixed Col-Gel films, it seems that only Col-promoted cell attachment (due to interactions of α2β1 with GxOGER) is happening for C2C12-α2+ cells as attachment was almost wholly insensitive to the presence of cRGD. This result suggests that Gel in the mixture with Col does not significantly influence the nature of the integrin specific binding of cells expressing both Col and Gel-recognition integrins. The adhesion of C2C12 parent cells on Gel films confirms the importance of the conformation and hence the appropriate exposure of the binding ligands in producing integrin-mediated cell-substrate interactions. The results show that in Gel films the configuration of the linear RGD motif was recognisable by cells (leading to cell adhesion), while in monolayer coated surfaces this ligand seems is apparently not detectable by cell surface integrins (no attachment, no spreading).

Crosslinking strongly decreases integrin-promoted cell binding to all films, which indicates that important cell recognition sequences, vital for cell-substrate interactions, were consumed in EDC-promoted crosslinking. These results are in agreement with our recent reports [15, 32, 44], which showed that carbodiimide treatment of collagenous materials may significantly decrease the content of carboxylic groups on glutamate and aspartate amino acid residues, leading to decrease of platelet attachments on highly crosslinked Col-based biomaterials.

### 4.3 Adhesion on scaffolds in comparison with films

The 3D scaffolds used for cell attachment tests have been previously characterised in terms of morphology, dissolution properties and swelling, which are important structural determinants of biological activity on protein matrices [6, 10]. Scaffold morphology, and especially pore size, influences not only 3D dimensional parameters, which control cell migration (as, for example, percolation diameter [6]), but also affects the specific surface and, as a consequence, the ligand density on scaffold struts available for cell binding [10]. During cell culture, swelling kinetics, and degradation rates control the degree of media uptake and stability of scaffold structure, respectively, which are likely to influence cell-substrate interactions. SEM analysis of scaffolds showed that crosslinking with EDC and/or the addition of Gel to Col had no significant effect on scaffold inner structure: all protein matrices used in cell experiments possessed a very similar morphology with homogeneous interconnected inner architecture and the pore diameters typically between 130–260 µm [15], these being suitable for the growth of myocytes, fibroblasts, and other cells [45, 46]. Swelling profiles and dissolution behaviour of all 100 % EDC XL scaffolds (from pure Col to pure Gel) were also comparable during the early stages of incubation (unpublished results): 3D constructs reached the maximum swelling after 1–2 h of soaking in aqueous media and all 100 % EDC XL samples exhibited similar structural stability (during incubation period covering completely the duration of cell adhesion assays on scaffolds [15]). Due to similarity in scaffold inner architecture and in swelling/dissolution characteristics, the differences found in cell behaviour on scaffolds were attributed to changes in base protein (addition of Gel to Col) or to the consequence of EDC crosslinking but not to the differences in scaffold morphology or their physical properties.

The results of adhesion studies on scaffolds revealed that the addition of Gel to Col produced an effect on cell attachment on 3D matrices very similar to that found on films. However, there was a very substantial difference between cell adhesion profiles on 2D films and 3D scaffolds: only integrin mediated binding was a characteristic feature of films, while 3D scaffolds showed a high level of non-specific interactions for all compositions and cell lines. This non-specific (in presence of EDTA) adhesion on scaffolds increased with the extent of crosslinking and may be attributed to cell entrapment within scaffold struts. It is possible that EDTA promoted non-specific cell binding was also present in 2D films but was completely removed by a rigorous washing treatment applied to these systems after cell attachment. However, in scaffolds this non integrin-mediated cell bonding remained, even after extended washing procedure, as a result of the contribution of sponge-like architecture to the entrapment of weakly bound cells. It seems that this phenomenon is dependent on the degree of intra/inter-molecular bond formation in Col fibrils, promoted by EDC crosslinking. The level of this non-integrin-dependent attachment should be considered for the correct evaluation of the biological performance of 3D scaffolds, since it has been reported that non-specific cell-binding on biopolymer surfaces does not lead to further regenerative activity of TE cell-scaffolds constructs [10].

The studies on films and scaffolds show that the integrin-dependent cellular response was highly dependent on the specific cell type and on the nature and amount of the adhesion motifs on the substrate. It was demonstrated that chemical crosslinking via the carbodiimide procedure, which is widely used in scaffold design for the purpose of enhancing physical and mechanical properties, ablates Mg-dependent integrin-binding cell activity on samples with both 2D (films) and 3D (scaffolds) architectures. This effect of EDC-mediated crosslinking may be attributed to the consumption of carboxylic groups on glutamate and/or aspartate residues in the native Col and Gel sequences, these being crucial for cell-substrate interactions.

### 4.4 The most characteristic features of cell adhesion on surfaces, films, and scaffolds

The experimental approach based on a systematic increase of the complexity of substrate under study (from monolayer coatings to thin films and finally to 3D scaffolds) used in this work shows the potential for deconvoluting the influence of the chemical identity of cell-recognition sequences from the effect of the bulk material and dimensional properties (2D vs. 3D architecture) on the nature and extent of cell-substrate interactions on protein-derived materials. The results obtained may be summarized as shown in Table 2, where strong and weak points of each system (monolayers, films, and scaffolds) are emphasized.

**Table 2:** Analysis of cell binding on substrate with different dimensional architectures

Substrate studied	Influence on cell activity	Results		Recommendations
		Advantages	Disadvantages	
Monolayer coatings	Cell recognition sequences alone (nature, availability, conformation)	✓Reliable and rapid assessment of sensitivity of substrate to integrin recognition alone	✗Conformation of ligand may be affected by surface adsorption	✓Screening of a broad range of compositions but possible conformational changes should be considered
		✓High accuracy and reproducibility		
		✓No cell entrapment		
2D films	+Bulk material properties	✓Conformation of cell-reactive ligands not affected by interaction with surface	✗3D scaffolds morphology not taken into account	✓Simple and effective way of study of the cell-scale properties of scaffolds
				✓Assessment of different treatments (XL) on integrin promoted binding
		✓Model of scaffolds struts		
		✓No cell entrapment with rigorous washing routine		
3D scaffolds	+3D morphology	✓Effect of 3D morphology on active cell binding of protein formulations with different composition and XL status	✗High level of non-specific adhesion due to possible physical cell entrapment	✓Testing of cell activity in more realistic 3D environment
			✗Lower accuracy /reproducibility than on coatings/films	✓Cell entrapment should be considered in assessment of scaffold biological activity

## 5 Conclusions

Coated surfaces provide a reliable and rapid assessment of sensitivity of a molecular substrate to integrin recognition alone but the conformation and hence exposure of biological motifs may be compromised by their close interaction with the underlying surfaces, especially for the denatured Gel. The conformation of cell-reactive ligands is not affected by surface contact on films so that these 2D systems may provide a reliable way of screening a broad range of compositions and treatments such as crosslinking on integrin-specific cell binding. The adhesion on 3D scaffolds revealed that sponge-like morphology seems to be responsible for a high level of non-integrin specific interactions on crosslinked samples, which should be considered when assessing the biological activity of 3D substrates. By systematically altering the composition, crosslinking, and 2D or 3D architecture of the substrate we provide simple, but effective, means to assess separately the contribution of the effects of morphology, physical parameters, and chemistry (available binding sites) on the cell activity of protein-derived materials. This information is important in the effective design of optimised surface chemistries in scaffolds for tissue repair.
